# Effects of Different Hosts on Bacterial Communities of Parasitic Wasp *Nasonia vitripennis*

**DOI:** 10.3389/fmicb.2020.01435

**Published:** 2020-07-07

**Authors:** Ruxin Duan, Heng Xu, Shanshan Gao, Zheng Gao, Ningxin Wang

**Affiliations:** ^1^Shandong Provincial Key Laboratory for Biology of Vegetable Diseases and Insect Pests, Department of Entomology, College of Plant Protection, Shandong Agricultural University, Tai’an, China; ^2^College of Life Sciences, Shandong Agricultural University, Tai’an, China

**Keywords:** bacterial community, fly, horizontal transfer, *Nasonia vitripennis*, parasitism, *Wolbachia*

## Abstract

Parasitism is a special interspecific relationship in insects. Unlike most other ectoparasites, *Nasonia vitripennis* spend most of its life cycle (egg, larvae, pupae, and early adult stage) inside the pupae of flies, which is covered with hard puparium. Microbes play important roles in host development and help insect hosts to adapt to various environments. How the microbes of parasitic wasp respond to different fly hosts living in such close relationships motivated this investigation. In this study, we used *N. vitripennis* and three different fly pupa hosts (*Lucilia sericata*, *Sarcophaga marshalli*, and *Musca domestica*) to address this question, as well as to illustrate the potential transfer of bacteria through the trophic food chains. We found that *N. vitripennis* from different fly pupa hosts showed distinct microbiota, which means that the different fly hosts could affect the bacterial communities of their parasitic wasps. Some bacteria showed potential horizontal transfer through the trophic food chains, from the food through the fly to the parasitic wasp. We also found that the heritable endosymbiont *Wolbachia* could transferred from the fly host to the parasite and correlated with the bacterial communities of the corresponding parasitic wasps. Our findings provide new insight to the microbial interactions between parasite and host.

## Introduction

As the most abundant animals worldwide, insects benefit greatly from their symbionts, as they help the insects in developing different strategies to adapt to various environments. With the rapid development of high-throughput sequencing technology, the composition and roles of microorganisms associated with insects have aroused extensive interest in the scientific community.

Insect-associated microorganisms participate in many life processes of their hosts, such as nutrition and digestion (Baumann et al., 1995; Wernegreen, 2002; Zientz et al., 2004; Toh, 2005; Geiger et al., 2011), immune defense (Hedges et al., 2008; Mahadav et al., 2008; Teixeira et al., 2008), reproductive modifications (Stouthamer et al., 1999; Lawson et al., 2001), and pesticide resistance (Kikuchi et al., 2012). Some microbes also play vital roles in the coevolution and speciation of their host insects (Hosokawa et al., 2006; Gosalbes et al., 2010; Werren et al., 2010; Brucker and Bordenstein, 2013). As an example, the endosymbiont *Wolbachia* infects almost 65% of insects (Hilgenboecker et al., 2008) and helps in evolution of host reproductive strategies, such as cytoplasmic incompatibility, male killing, feminization, parthenogenesis, and even speciation (Bordenstein, 2003; Werren et al., 2008; Brucker and Bordenstein, 2012a; Gebiola et al., 2016; Shropshire and Bordenstein, 2016).

Many factors can affect the bacterial communities of insects: firstly, the species of insect hosts, which is known to shape their bacterial communities. For example, the microbial communities of honey bees and bumble bees exhibit similar patterns as the clustering of the host (Martinson et al., 2011; Koch et al., 2013). Secondly, diet can affect the hosts’ bacterial diversity. Previous studies have found that the composition of intestinal microorganisms in larvae of *Helicoverpa armigera* (Lepidoptera, Noctuidae) is similar to that of the plant community, indicating that food is an important source of intestinal microorganisms (Priya et al., 2012). According to a study on *Monochamus alternatus* (Coleoptera, Cerambycidae), *Enterococcus* is the dominant bacterium in the intestinal tract of larvae feeding on natural food, while *Lactococcus* is the dominant bacterium in the intestinal tract of larvae feeding on artificial feed (Kim et al., 2017). Distantly related hosts that live in close symbiotic relationship can also maintain similar microbial communities. With varying degrees of nest sharing between them, *Megalomyrmex* social parasites (Solenopsidini) and their fungus-growing ant hosts from the genera *Cyphomyrmex*, *Trachymyrmex*, and *Sericomyrmex* share symbiont bacteria (Liberti et al., 2015). Thirdly, spatial and temporal distributions of the host could influence their bacterial compositions (Zhang and Blackwell, 2002; Falush et al., 2003; Hedlund and Staley, 2004; Papke and Ward, 2004; Martiny et al., 2006). The bacterial structure of termites shows significant differences between diverse sampling sites (Hongoh et al., 2005).

*Nasonia vitripennis* (Hymenoptera, Pteromalidae) is an important parasitoid whose female wasp stings, injects venom, and lays eggs in many different fly pupae, where their eggs, larvae, pupae, and early-stage adults are developed. *N. vitripennis* usually lives on species of the family Calliphoridae, Muscidae, and Sarcophagidae. Because of its short generation time, large offspring production, and easy rearing, *Nasonia* has emerged as a model organism for developmental and evolutionary genetics (Werren and Loehlin, 2009), as well as a research model for host–microbial community interaction studies (Dittmer et al., 2016).

Parasitism is a special symbiotic relationship in insects. Unlike other ectoparasitic wasps that usually live on the surface of their hosts, *N. vitripennis* spends most of its life cycle inside fly species, which are covered with puparium; thus, it lives in an enclosed environment. The microbiota from both fly species and the wasps may have more opportunities to communicate due to such an enclosed environment (Lynch, 2015). The microbial communities of *N. vitripennis* have been previously reported (Brooks et al., 2016; Jessica et al., 2016). However, the response of the microbial community composition of the wasps to different hosts in such an enclosed environment is still unknown. In this study, we used *N. vitripennis* and three different fly hosts (*Lucilia sericata*, *Sarcophaga marshalli*, and *Musca domestica*) with different food resources to address the following issues: (1) how the different hosts affect the bacterial communities of *N. vitripennis*, (2) whether there is bacterial transfer through food chains, and (3) whether the existence of the endosymbiont *Wolbachia* in fly species could affect the microbiota of the parasitic wasp.

## Materials and Methods

### Experimental Insects

**FIGURE 1:**
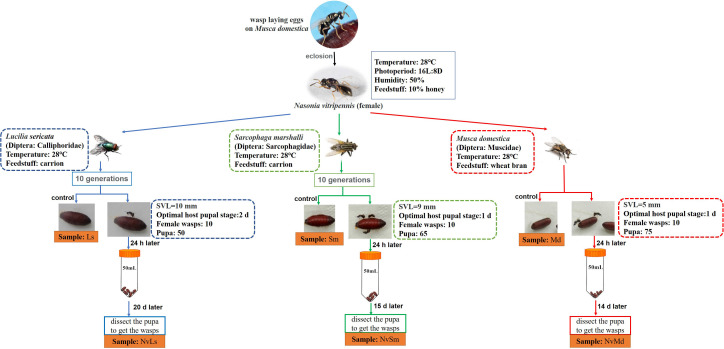
Feeding conditions of experimental insects in laboratory and acquisition of experimental samples. Snout–vent length (SVL) represents the body length of the fly species.

Three different fly hosts, *L. sericata* (Ls, Diptera: Calliphoridae), *S. marshalli* (Sm, Diptera: Sarcophagidae), and *M. domestica* (Md, Diptera: Muscidae), were trapped at the campus of Shandong Agricultural University, China, and then have been kept in the laboratory since 2012. Fly traps were baited with household pork to obtain eggs of the carnivorous *L. sericata* and *S. marshalli*, as well as wheat bran mixed with water to obtain eggs of *M. domestica*. Laboratory rearings were performed with pork carrion (Ca) for the carnivorous flies (*L. sericata* and *S. marshalli*), whereas *M. domestica* was reared on wheat bran (Wb). For larval development rearing was done at 28°C, 50% relative humidity (RH) and 16:8 h (L:D) photoperiod. All fly adults were provided with a 10% honey solution (in water) provided *ad libitum* (Meister, 1962). *N. vitripennis* was routinely reared on *M. domestica*, at the same rearing conditions as descried above. Microbial diversity assays were performed starting from this lab strain, as indicated in Figure 1. Briefly, coetaneous *N. vitripennis* females were split into three batches. Each batch was further reared on *L. sericata* (NvLs), *S. marshalli* (NvSm), or *M. domestica* (NvMd) pupae for 10 generations. After 10 generations, parasitized hosts (1-day-old pupae) were isolated in 50-ml tubes to allow parasitoid development, and unparasitized hosts were used as controls, as indicated in Figure 1.

### DNA Extraction, PCR Amplification, and Sequencing

Regardless of gender, fresh fly species at the pupal stage (1-day-old) were collected for DNA extraction, although sex could have an impact on microbial communities (Han et al., 2017). Twenty *M. domestica* pupae, 10 *S. marshalli* pupae, and 10 *L. sericata* pupae were selected (members of each fly type were considered as one sample), while more than 100 *N. vitripennis* individuals were collected before emerging from each of the fly hosts. The fresh fly pupa hosts and parasitic wasps were sterilized in 75% ethanol for 1 min and washed in sterile water for 30 s three times. After sterilization, the samples were collected in 1.5-ml centrifuge tubes and stored in liquid nitrogen. In order to avoid bacterial contamination outside of the puparium, which could affect the bacterial composition of the wasps, after 12 days of the parasite inside the pupa, the puparium was broken with sterilized tweezers and knives. Further, *N. vitripennis* was transferred to sterilized 1.5-ml centrifuge tubes with small holes to maintain air circulation for the wasps to develop. After 2 days, the wasps were sterilized in 75% ethanol for 1 min and washed in sterile water three times. About 100 individuals were collected in a 1.5-ml centrifuge tube and stored in liquid nitrogen for DNA extraction. Total DNA was extracted from the fly species at the pupal stage and parasitic wasps from different fly hosts, carrion meat, and wheat bran using the OMEGA Soil DNA Extraction Kit. The extracted DNA was amplified by PCR using the bacterial 338F (5′-ACTCCTACGGGAGGCAGCAG-3′)-806R (5′-GGACTACHVGGGTWTCTAAT-3′) primer set targeting the 16S rRNA gene. PCR conditions were as follows: pre-denaturation at 94°C for 5 min; 35 cycles of denaturation at 94°C for 40 s, annealing at 45–59°C (adjusted according to different primers) for 30–60 s, and an extension at 72°C for 1 min; and a final extension for 10 min at 72°C. The qualified DNA samples were selected and sequenced on the Illumina MiSeq platform of Majorbio Bio-Pharm Technology Co., Ltd., Shanghai, China.

The gene coding for *Wolbachia* surface protein (*wsp* gene) was detected using the *Wolbachia*-specific primer set of 81F (5′-TGGTCCAATAAGTGATGAAGAAAC-3′) and 691R (5′-AAAAATTAAACGCTACTCCA-3′), and the amplification profiles all followed Xiao et al. (2012). It was observed that *L. sericata* and *M. domestica* were not infected with *Wolbachia*, while *S. marshalli* was divided into two groups: one group was infected with *Wolbachia*, while the other group was *Wolbachia* free.

### Bioinformatics and Statistical Analyses

MiSeq sequencing provided double-ended sequence data. First, according to the overlap between paired-end (PE) sequencing reads, pairs of reads were spliced (merge) into a sequence, the reads were filtered based on their quality, and the results were merged by using QIIME2 (Bolyen et al., 2019). The quality filtering standards for sequential reads are as follows. First, the reads with a mass value of less than 20 were filtered, and a 50-bp window was set. If the average mass value in the window is less than 20, the back-end bases were truncated from the window. Reads below 50 bp after quality control were filtered to remove the reads containing N bases. Then, according to the overlap relationship between PE reads, paired reads were spliced (merged) into a sequence, and the minimum overlap length was 10 bp. Then, the maximum error ratio allowed in the overlap area of the mosaicing sequence is 0.2, and the nonconforming sequence is screened. Finally, samples were differentiated according to the barcode and primer at both ends of the sequence, and the sequence direction was adjusted. The allowable mismatches of the barcode were 0, and the maximum primer mismatches were 2. According to the sequence of the two ends of the barcode and the primer sequence, the valid sequences were distinguished from the sample. Then the sequence orientation was corrected, and the optimized data were acquired. The non-repetitive sequences were extracted from the optimized sequences, which could reduce the computational complexity of the intermediate process, the single sequences without duplication were removed, and operational taxonomic units (OTUs) based on the non-repetitive sequences (excluding the single sequence) were clustered according to 97% similarity by using UCHIME. During the clustering process, chimera sequences were removed, and the representative sequences of OTUs were obtained. All the optimized sequences were mapped to the OTU representative sequence, and the sequences with a similarity of more than 97% were selected to generate the OTU table. In order to obtain the information of the species corresponding to each OTU, the RDP classifier Bayesian algorithm was used to analyze 97% similarity of the OTU representative sequence against the SILVA ribosomal RNA gene database using a confidence threshold of 70%.

Based on 97% similarity of the OTUs or other taxonomic levels, the diversity of random sampling in the form of diversity index was calculated using mothur. Alpha diversity and beta diversity were calculated by GmT (Galaxy mothur Toolset) and QIIME2, respectively (Hiltemann et al., 2018; Bolyen et al., 2019). In order to study the similarity or differences in the bacterial composition of the samples, the number of common and unique OTUs in the samples was counted. Based on UniFrac coupled with principal coordinate analysis (PCoA) for all the samples, PCoA and Venn graphs were plotted using the R package. A heatmap plot for abundance analysis was made by using the R package. According to the classification of the composition of the samples and different grouping criteria for linear discriminant analysis (LDA), LDA effect size (LEfSe) found a significant difference in the sample division of the community or species. Differences between populations were analyzed by using one-way ANOVA. Excel was used to show the horizontal transfer of bacteria through the tertiary food chain food–host–parasitic wasp. Information about the species within or between groups was obtained by using the NetworkX. The Network Analysis Toolkit was used to calculate the node degree distribution, the diameter and the average shortest path of the network, the degree of connectivity, and closeness centrality (Hagberg et al., 2008). In Cytoscape 2.8.3, the layout was set as spring embedded, and nodes were used as connection points to reflect the connection between the sample and OTUs and to make a network diagram (Shannon et al., 2003). In order to explore the influence of *Wolbachia* on other bacteria, SPSS 22.0 was used to calculate the Pearson rank correlation coefficient, and Gephi 0.9.2 (Bickel, 2003) was used for figures. In addition, we took “fly species–parasitic wasp” and “carrion/wheat bran–fly species–parasitic wasp” as the whole, and the bacteria that were shared in host, parasitic wasp, and food in the food chain were figured out using Excel and iTOL software (Letunic and Bork, 2019). FastTree was used to construct the phylogenetic tree based on the maximum likelihood method by selecting the sequences corresponding to OTUs or a class of classified information at different levels. According to the cluster of orthologous groups of proteins (COG) database, the descriptions of each COG and its function were analyzed based on the eggNOG database (Huerta-Cepas et al., 2019). In addition, PICRUSt was used to obtain different levels of metabolic pathway information, as well as the abundance table at each level (Langille et al., 2013).

## Results

### *Nasonia vitripennis* Microbiota Profiles in Different Host Species

A total of 754,311 16S rRNA gene sequences were obtained (accession number PRJNA479943). There were 651 OTUs, which were clustered based on 97% 16S rRNA similarity and belonged to 23 prokaryotic phyla. Rarefaction curves showed that all the samples showed enough sequencing depth to represent their bacterial diversity (Supplementary Figure S1).

**FIGURE 2:**
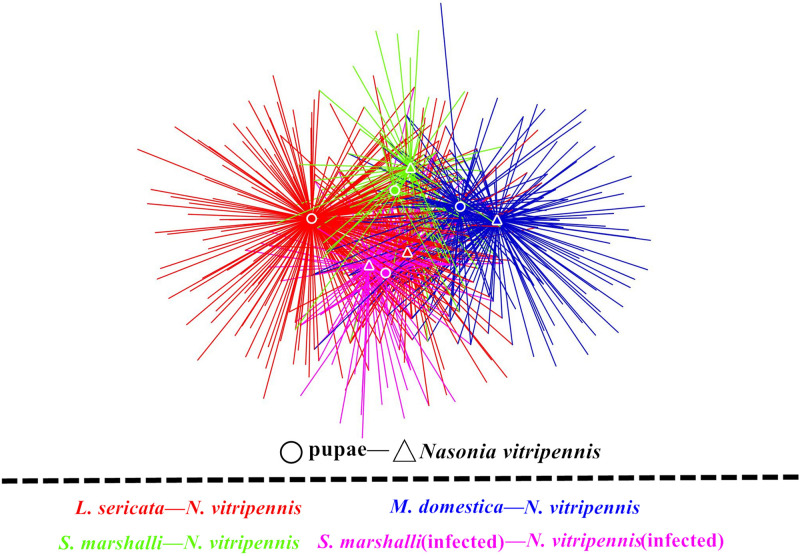
Microbiota network of different fly hosts and their corresponding *N. vitripennis*. Fly hosts and *N. vitripennis* are shown as circles and triangles, respectively. Different colors represent the four categories of specifically associated host and parasitic species. Colors red, yellow, green, and purple represent *L. sericata*, *M. domestica*, *Wolbachia-*free *S. marshalli*, and *Wolbachia-*infected *S. marshalli* and their corresponding *N. vitripennis*, respectively.

Interestingly, the network showed a significant microbiota clustering of *N. vitripennis* and their corresponding fly hosts rather than the bacterial clustering based on fly species (Figure 2). *M. domestica* and *Wolbachia*-infected *S. marshalli* (SmIn) and *Wolbachia*-free *S. marshalli* (Sm) showed a closer relationship and more intersections in terms of bacterial community composition with their corresponding parasitic wasps than did *L. sericata* and its parasitic *N. vitripennis*; both showed the maximum number of unique OTUs.

### Effect of Different Fly Hosts on Microbial Community of Parasitic Wasps

**FIGURE 3:**
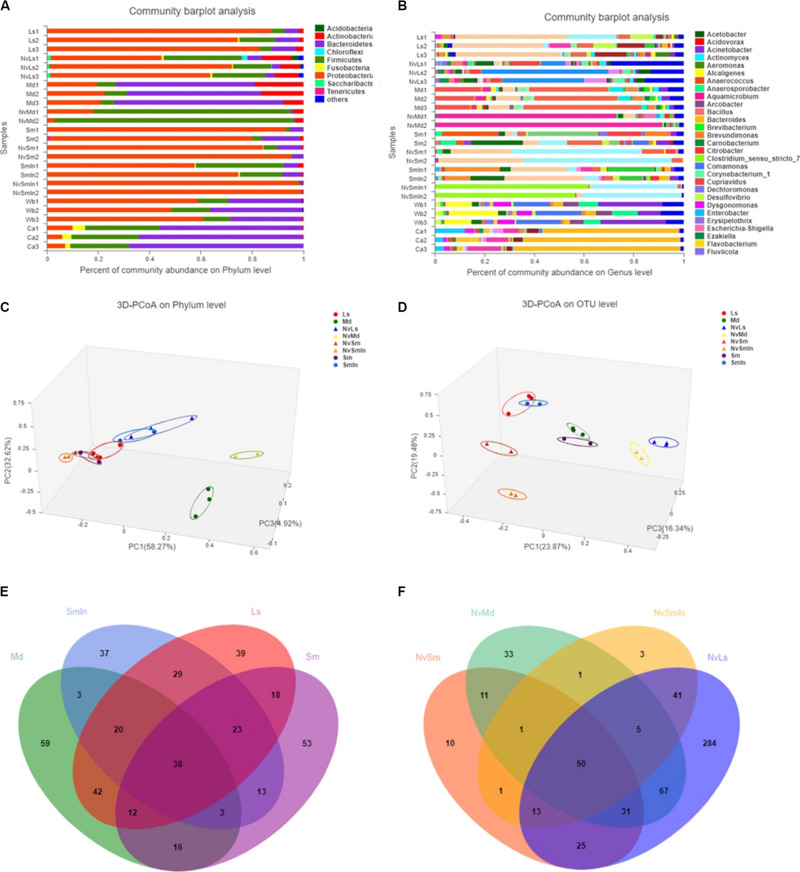
Bacterial composition analysis. Column plots of communities of different samples at phylum level **(A)** and genus level **(B)**. The length of the columns represents the proportion of species. Bray–Curtis principal coordinate analysis (PCoA) of microbial communities at phylum level **(C)** and OTU level **(D)**. PC1 and PC2 are two principal coordinate components. Venn diagram at OTU level of fly hosts (*L. sericata*, *M. domestica*, *Wolbachia-*free *S. marshalli*, and *Wolbachia-*infected *S. marshalli*) **(E)** and corresponding *N. vitripennis* **(F)**. Different colors represent different samples; the numbers of overlaps and folds represent the shared and the unique species numbers, respectively.

Wasps and their fly hosts showed different bacterial communities. At the phylum level, Proteobacteria was predominant in both *L. sericata* and *S. marshalli* whose adults were fed with carrion, while Bacteroidetes and Proteobacteria shared the predominant position in *M. domestica* whose adults were fed with bran. For *N. vitripennis*, Proteobacteria was predominant in wasps with *L. sericata* (NvLs) and *S. marshalli* (NvSm) as hosts, while Firmicutes was dominant in wasps with *M. domestica* (NvMd) as the host (Figure 3A). At the genus level, the differences were more defined (Figure 3B). *Providencia* (41.28%) and *Myroides* (37.61%) were the predominant genera in *L. sericata* and *M. domestica*, respectively. For *Wolbachia*-free *S. marshalli* (Sm), *Massilia* (16.19%), and *Sphingobium* (12.85%) were the predominant genera, while for *Wolbachia*-infected *S. marshalli* (SmIn), *Providencia* (27.27%) and *Vagococcus* (16.13%) were the predominant genera, although there was no evident difference at the phylum level due to the presence or absence of *Wolbachia*. The bacterial communities of the parasitic wasps from different hosts were quite interesting. *Gluconobacter* (31.24%) and *Staphylococcus* (81.47%) were the dominant bacterial genera in wasps whose hosts were *L. sericata* (NvLs) and *M. domestica* (NvMd), respectively. *Proteus* (56.03%) and *Wolbachia* (59.17%) were the dominant genera in *N. vitripennis* whose hosts were *Wolbachia*-free (Sm) and *Wolbachia*-infected *S. marshalli* (SmIn), respectively (Figure 3B).

Bacteroidetes was the predominant bacteria in carrion, which was used as food for carnivorous insects *L. sericata* and *S. marshalli*, while Proteobacteria was the predominant bacteria in wheat bran, which was used as food for the phytophagous fly *M. domestica* (Figure 3A). The bacterial community of *N. vitripennis* was quite different from its fly hosts based on PCoA (Figures 3C,D and Supplementary Figure S2). Chao and Ace indices showed that there were significant differences in the bacterial diversity index between different samples (Supplementary Table S1). The bacterial diversity of *N. vitripennis* with *L. sericata* (NvLs) as host was significantly higher than that of the other samples. A rank–abundance graph showed that the curve length and span of *N. vitripennis* with *L. sericata* (NvLs) as host were higher than those of the other samples, indicating that the species richness and species uniformity of this sample were the highest (Supplementary Figure S3).

**FIGURE 4:**
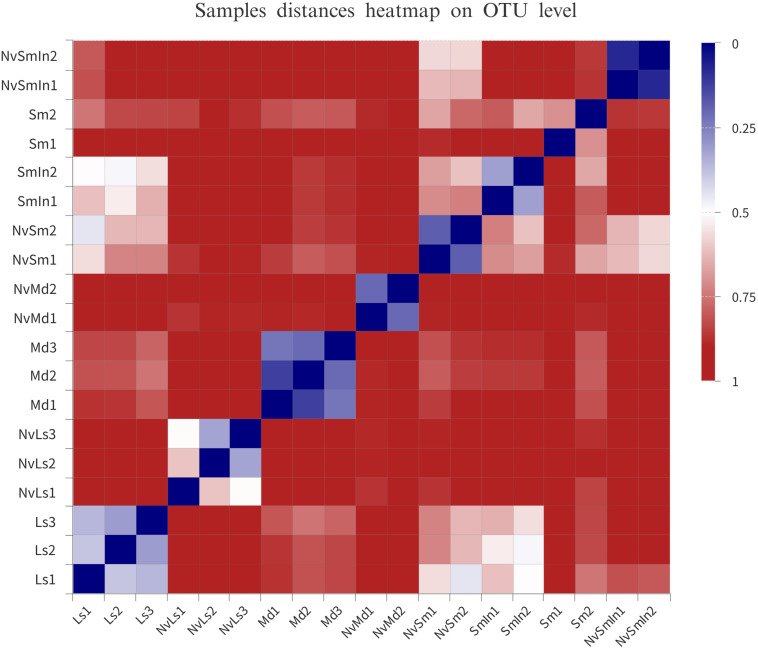
Distance heatmap based on OTU level of the samples. The distance between samples is represented by different color gradients (the value represented by the color gradient on the right of the figure).

The bacterial hierarchical clustering of the samples did not occur based on species. *M. domestica* and its corresponding *N. vitripennis* (NvMd) were close to wheat bran in a distance similar to *L. sericata* and its corresponding *N. vitripennis* (NvLs). The parasitic wasps hosted by *Wolbachia*-infected (SmIn) and *Wolbachia*-free *S. marshalli* (Sm) were clustered together (Figure 4).

There were 221 and 193 OTUs detected in *L. sericata* and *M. domestica*, respectively, and there were 166 and 176 OTUs in *Wolbachia*-infected (SmIn) and *Wolbachia*-free *S. marshalli* (Sm), respectively (Figure 3E). *Acinetobacter*, *Bacillus*, *Citrobacter*, *Lactococcus*, *Morganella*, and *Providencia* were common in all types of fly species (Figure 3E). *Massilia*, *Bacillus*, *Serratia*, *Providencia*, *Lactococcus*, *Staphylococcus*, *Morganella*, *Proteus*, *Bacteroides*, *Enterobacter*, and *Myroides* were common OTUs in *N. vitripennis* hosted by all the fly species (Figure 3F). The bacterial diversity of *N. vitripennis* with *L. sericata* (NvLs) as host was also higher than that of *N. vitripennis* inhabiting other hosts. A total of 516 and 199 OTUs were detected in *N. vitripennis* with *L. sericata* (NvLs) and *M. domestica* (NvMd) as hosts, respectively, while there were 142 and 115 OTUs in *N. vitripennis* with *Wolbachia*-free (NvSm) and *Wolbachia*-infected *S. marshalli* (NvSmIn) as hosts, respectively (Figure 3F). The results showed that bacterial diversity of the same *N. vitripennis* with a different fly host was significantly different.

### Potential Bacterial Transfer via Food–Fly–Wasp Tertiary Food Chain

**FIGURE 5:**
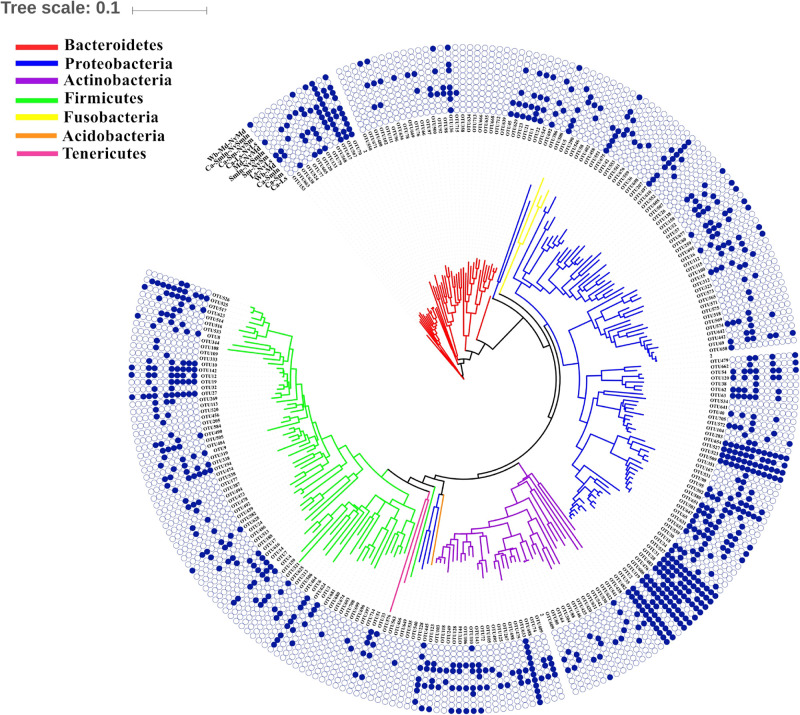
Shared bacteria of different food chains (food–pupae–wasps or pupae–wasps). The phylogenetic tree was constructed by the top 50 OTUs in each sample (internal). The blue solid circle represents the presence of the bacterial OTUs in the food chain, and the hollow circle represents the absence of bacterial OTUs in the food chain. For example, OTU153 showed that it was able to pass through the food chain of the wheat bran–*M. domestica* pupae–its corresponding *N. vitripennis*. In addition, it was also able to pass from *L. sericata* pupae to *N. vitripennis*.

In this study, the parasitic wasp *N. vitripennis* was grown in three different fly hosts. *S. marshalli* and *L. sericata* were fed with carrion, while *M. domestica* was fed with wheat bran; thus, the food–pupae–wasp tertiary food chain and the pupae–wasp food chain system were good models to study the potential bacterial transmission (Figure 5 and Supplementary Table S2).

There were some unique OTUs found in different food–pupae–wasp tertiary food chains. OTU20 (*Myroides*) was found only in the food chain beginning with carrion (carrion–*L. sericata*/*S. marshalli*–*N. vitripennis*), while OTU102 (*Sphingobacterium*), OTU111 (*Sphingobacterium*), OTU92 (*Sphingobacterium*), OTU104 (*Pseudomonas*), OTU106 (*Microbacterium*), OTU146 (*Actinomyces*), and OTU662 (*Stenotrophomonas*) were unique to food chains that began with wheat bran (wheat bran–*M. domestica*–*N. vitripennis*); hence, these bacteria were probably transferred from food to fly species to *N. vitripennis.*

Many unique bacteria only existed in the fly species at the pupal stage to parasitic wasp food chains and were not found in food to fly food chains. Most unique OTUs were found in the *L. sericata*–*N. vitripennis* food chain and confirmed the possibility of being transmitted from fly species to *N. vitripennis* (Figure 5 and Supplementary Table S2). Some of the unique bacteria only existed in one of the two food chain systems, from food to fly species or from fly to wasps; these were rare bacteria appearing at a low abundance.

### Effects of *Wolbachia* on Host Microbiota

**FIGURE 6:**
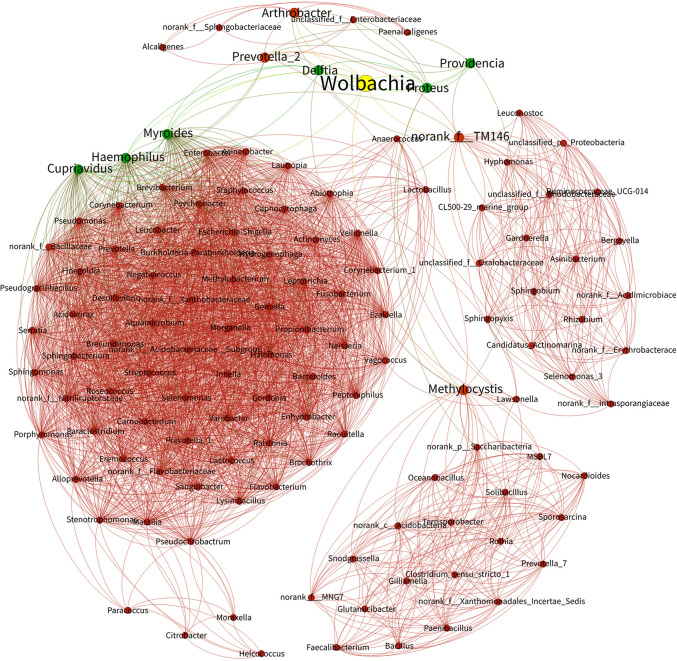
Bacterial networks from *Wolbachia*-infected and *Wolbachia*-free *N. vitripennis*. The Spearman rank correlation coefficient was calculated to reflect the correlation between bacterial species. Red and green lines represent positive and negative correlations, respectively.

Taking all the bacteria from *Wolbachia*-infected (NvSmIn) and *Wolbachia*-free *N. vitripennis* (NvSm) into account, certain large coenobium was observed in this study. The coenobium, including *Proteus*, *Myroides*, *Haemophilus*, *Delftia*, *Arthrobacter*, *Cupriavidus*, *Providencia*, *Methylocystis*, *Prevotella*_2, and norank_f_TM146 showed a direct relationship with *Wolbachia* (Figure 6). Except in the cases of *Prevotella*_2, *Methylocystis*, *Arthrobacter*, and norank_f_TM146, the Spearman correlation coefficient between other genera and *Wolbachia* showed negative correlations, suggesting that *Wolbachia* and these genera may be in a competitive relationship (Figure 6 and Table 1).

**TABLE 1:** Spearman correlation coefficients of *Wolbachia* and some related bacteria.

	*Wolbachia* correlation coefficient	COR	Significance test
*Prevotella*_2	0.998007	P	*p* < 0.05
*Providencia*	−0.967822	N	*p* < 0.05
*Methylocystis*	0.851299	P	*p* < 0.05
*Proteus*	−0.95845	N	*p* < 0.05
*Myroides*	−0.873062	N	*p* < 0.05
norank_f_TM146	0.875827	P	*p* < 0.05
*Haemophilus*	−0.81487	N	*p* < 0.05
*Delftia*	−0.998007	N	*p* < 0.05
*Arthrobacter*	0.880868	P	*p* < 0.05
*Cupriavidus*	−0.902732	N	*p* < 0.05

*COR, correlation; P, positive correlation; N, negative correlation; *p* < 0.05 indicates significant difference.*

### Function Prediction Based on 16S rRNA Gene Sequence

**FIGURE 7:**
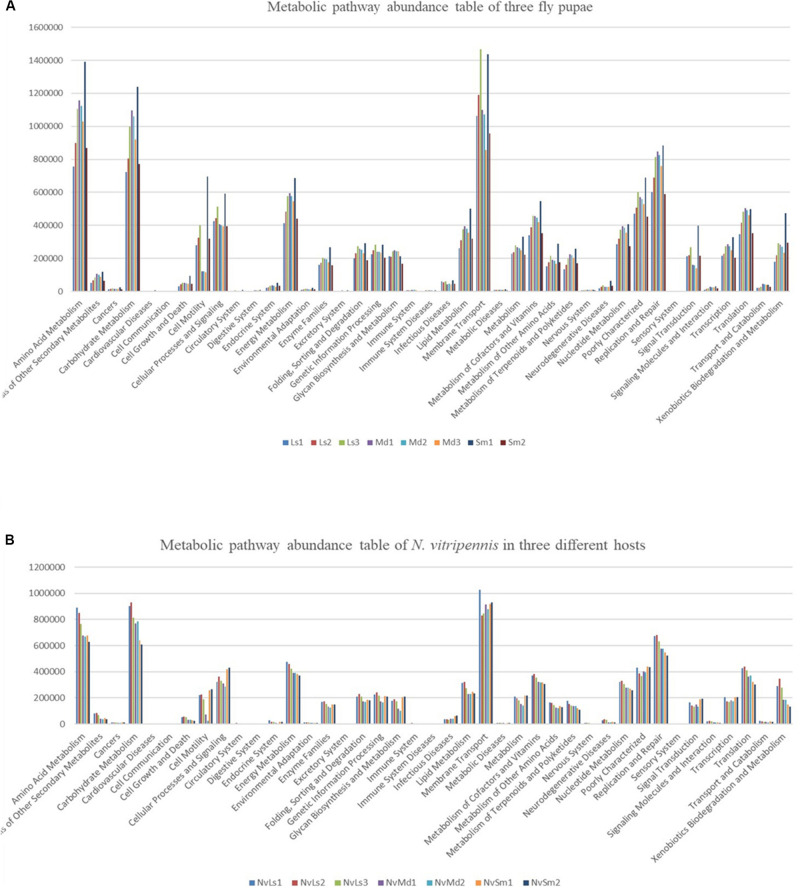
KEGG functional secondary classification of the three different fly pupa hosts **(A)** and their corresponding *N. vitripennis* **(B)**.

**FIGURE 8:**
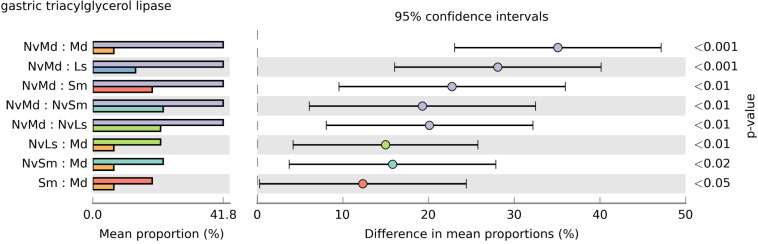
The significance test of gastric triacylglycerol lipase expression abundance based on Welch’s *t*-test and a two-sided test. The dot color is shown the same as the group color with large expression abundance. Only results with a *p*-value < 0.05 are shown.

All the bacterial KEGG metabolic pathways were mainly classified into amino acid metabolism, carbohydrate metabolism, replication and repair, energy metabolism, glycan biosynthesis and metabolism, enzyme families, membrane transport, etc. (Figure 7). Gastric triacylglycerol lipase helps insects in digestion of fat. Comparison of the differences in gastric triacylglycerol lipase expression in different fly pupae and their corresponding *N. vitripennis* showed that the lipase expression in carnivorous *N. vitripennis* was significantly higher than that in the phytophagous *M. domestica*, which was fed with wheat bran. Moreover, the lipase expression in *S. marshalli* was significantly higher than that in *M. domestica* (Figure 8). Although PICRUSt was deemed not suitable for nonhuman microbiomes (Langille et al., 2013), we thought it is believable in our results.

## Discussion

Parasitism is a symbiotic insect relationship wherein the parasite consumes its host as nutrition for development, unlike commensalism and mutualism. *Nasonia* is a special ectoparasite with development of more than one wasp in a single fly pupa (brood size in *Nasonia* ranges from 3 to 46 per host pupa); they inject their eggs through the puparium of the fly host, where the eggs hatch and then develop into larvae, pupae, and adults. Unlike other ectoparasitoids that usually live on the surface of their hosts, living under the cover of puparium makes *Nasonia* and its fly hosts good systems to study the interactions between insect parasites and their hosts from different perspectives.

Our results demonstrate that different hosts can affect the bacterial communities of parasitic wasps. Species is the most contributing determinant in bacterial composition of different organisms, which was confirmed by our results. Although fly food, parasitic wasps, and fly hosts had some common bacteria, such as *Raoultella*, *Pseudomonas*, *Providencia*, *Myroides*, *Lactococcus*, *Acinetobacter*, and *Corynebacterium*, they also showed their unique microbiota composition pattern. However, the microbiota of the parasitic wasp *N. vitripennis* was shaped by its corresponding host doubtlessly. From the microbiota networks of the three fly pupae and their corresponding parasitic wasps, *M. domestica* and *Wolbachia*-infected and *Wolbachia*-free *S. marshalli* showed a closer relationship and more intersections with their corresponding parasitic wasps than *L. sericata* and its parasitic wasps; both showed many unique OTUs, individually (Figure 2). This phenomenon needs to be investigated in our next study. Besides organism species, diet and spatial environment are also important factors that influence microbiota of an organism. It is reported that dietary specialization in mutualistic acacia–ants affects the relative abundance of the host-associated bacteria (Rubin et al., 2019). For our parasitic wasps, fly species were not only the sole diet source from egg to adult but also their closed living environment. The unique life history of the ectoparasitic wasp *Nasonia* also makes it a good model to study the microecology of parasite and hosts.

Different fly hosts and their corresponding *N. vitripennis* showed different core microbiota. *Providencia*, *Myroides*, *Massilia*, and *Sphingobium* were predominant in *L. sericata*, *M. domestica*, *Wolbachia*-free *S. marshalli*, and *Wolbachia*-infected *S. marshalli* pupae, respectively. On the other hand, bacterial communities of the same parasitic wasps from different hosts were quite interesting. *Gluconobacter*, *Staphylococcus*, *Proteus*, and *Wolbachia* were the dominant bacteria genera in wasps whose hosts were *L. sericata*, *M. domestica*, *Wolbachia*-free *S. marshalli*, and *Wolbachia*-infected *S. marshalli*, respectively. Thus, the same parasitic wasp with different hosts showed different dominant bacteria.

Moreover, some of the bacteria mentioned above were also the dominant bacteria in one or more samples. For example, *Pseudomonas* was one of the most abundant bacteria in *Wolbachia*-free *S. marshalli*, and *Myroides* was one of the most abundant bacteria in *M. domestica*, which are in accordance with results from a previous study by Zurek and Nayduch (2016). However, *Providencia* abundantly existed not only in *L. sericata* but also in *Wolbachia*-infected *S. marshalli*. These bacteria were the core bacteria in these samples and had important regulating effects on the physiological activity of the host. Even though there was a close relationship between the parasitic wasps and their fly pupa hosts, as *N. vitripennis* spends almost its whole life in the fly species, the bacterial composition of the pupae hosts and *N. vitripennis* showed significant differences.

Different fly hosts showed significant effects on the bacterial diversity of the parasitic wasp *N. vitripennis.* For this special relationship in animals, parasitism means one can get benefit from the other, such as a developmental environment and nutrition. In case of *N. vitripennis*, its eggs, larvae, pupae, and young adults all developed inside the fly species at the pupal stage. Three different host sources of *N. vitripennis* showed different bacterial compositions. *Gluconobacter* and *Staphylococcus* were the dominant bacteria in *N. vitripennis* from *L. sericata* and *M. domestica*, respectively. *Proteus* and *Providencia* were the dominant bacteria in *N. vitripennis* with *Wolbachia*-free *S. marshalli* as host, while *Wolbachia* and *Proteus* were the dominant bacteria in *N. vitripennis* with *Wolbachia*-infected *S. marshalli* as host (Supplementary Figure S7 and Supplementary Table S3). Thus, different fly hosts shaped the bacterial diversity of their parasitic wasps. Brucker and Bordenstein (2012b) found that three species of *Nasonia* wasps, whose host was *Sarcophaga bullata*, were dominated by *Providencia*. Two possible reasons could explain this difference when compared to our results. Firstly, according to our findings, the hosts could affect the microbiota of parasitic wasps, and because different fly hosts were chosen, *N. vitripennis* from different hosts showed varied bacterial compositions. Secondly, different sequencing methods were used in both studies: Brucker used the first-generation sequencing method, while we used the high-through sequencing method, which can find trace bacteria; hence, our results should be more comprehensive. It is worth noting that the rearing temperature of *N. vitripennis* was different. Temperature can affect the microbiome of the host. Brucker used 25°C to rear the wasp, while we used 28°C in this study, so temperature is another possible reason for the microbial difference.

We also tried to predict a potential horizontal transfer of bacteria through secondary or tertiary food chains. *N. vitripennis* inhabiting *S. marshalli* were infected with *Wolbachia*, and their corresponding *N. vitripennis* were infected with *Wolbachia* too, while when the hosts were not infected with *Wolbachia*, the parasitic wasps were also *Wolbachia* free; this phenomenon is substantial evidence for horizontal transfer of bacteria, which was validated by Brucker and Bordenstein (2012b). Taking the wheat bran-*M. domestica-N. vitripennis* food chain as an example, OTU102 (*Sphingobacterium*), OTU111 (*Sphingobacterium*), OTU92 (*Sphingobacterium*), OTU104 (*Pseudomonas*), OTU106 (*Microbacterium*), OTU146 (*Actinomyces*), and OTU662 (*Stenotrophomonas*) were found in all three, wheat bran, *M. domestica*, and *N. vitripennis*, which suggested the potential horizontal transfer from food to fly and to the parasitic wasp. These food chains are a good model to investigate horizontal transfer of bacteria through a special parasitic relationship. Some trace bacteria were also observed in this study that may play important metabolic functions in their hosts, which were shared by *N. vitripennis* and their corresponding fly species. While providing substantial insights on the identity, structure, and taxonomic overlap of the microbiota in different food chains, our comparative results could not provide conclusive evidence for the underlying transmission mechanisms of every bacterial symbiont, because some may be shared in nature. To illuminate and confirm the transmission mechanisms, experiments are required in the future.

Endosymbiont *Wolbachia* is famous for various reproductive manipulations and fitness effects on their hosts. *Wolbachia* can not only influence the hosts’ mitochondrial DNA (Xiao et al., 2012) but also can alter hosts’ microbiota composition, for example, in the terrestrial isopod *Armadillidium vulgare* (Isopoda, Oniscoidea) (Dittmer and Bouchon, 2018) and in adult *Aedes aegypti* (Diptera, Culicidae) mosquitoes (Audsley et al., 2018). In our study, we found both competitive and cooperative relationships between the microbiota. The existence of *Wolbachia* influenced the presence and abundance of many bacterial taxa within each host population, possibly due to competitive interactions. *Proteus*, *Myroides*, *Haemophilus*, *Delftia*, *Arthrobacter*, *Cupriavidus*, *Providencia*, *Methylocystis*, *Prevotella*_2, and norank_f_TM146 showed symbiotic or competitive relationships with *Wolbachia*. The microbiota was shaped by interactions not only between the insect hosts and its symbionts but also between the different members of the symbiotic communities (Mouton et al., 2004; Oliver et al., 2006). Competition between *Wolbachia* and other microorganisms for resources and space in the shared host environments decreased the abundance of some bacteria.

The parasitic wasps and fly hosts are good models to study parasitic interactions from different perspectives. Detailed research on this issues, like the role of bacteria behind successful survival of wasps in new hosts, needs our attention.

## Data Availability Statement

The datasets generated for this study have been uploaded to GenBank by the accession number PRJNA479943.

## Author Contributions

NW conceived the idea of study and designed the methodology. HX prepared all the experimental insect samples. RD, HX, SG, ZG, and NW participated in data analysis and wrote sections of the manuscript. All authors contributed to the article and approved the submitted version.

## Conflict of Interest

The authors declare that the research was conducted in the absence of any commercial or financial relationships that could be construed as a potential conflict of interest.

## Supplementary Material

FIGURE S1 Sparse curve analysis of the samples.

FIGURE S2 Shannon index at OUT level of the samples.

FIGURE S3 Rank-abundance curves at OTU level of the samples.

FIGURE S4 Relationship between Circos samples and species which can reflect the distribution ratio of dominant species in each sample through a visual circle diagram.

FIGURE S5 Heatmap analysis of the top 50 species abundance in all the samples at the genus level.

FIGURE S6 Cyclic phylogenetic tree of the top 50 species abundance in all samples at the genus level.

FIGURE S7 Student’s *t*-test bar plot on Genus level between two samples.

TABLE S1 Alpha diversity index table of all the samples.

TABLE S2 Species abundance of partial OTUs in all samples.

TABLE S3 Statistical table of difference test between two groups of samples.
