# Supplementary figures and images for: Effects of Different Hosts on Bacterial Communities of Parasitic Wasp Nasonia vitripennis
Source: Front Microbiol. 2020 Jul 7;11:1435. doi: 10.3389/fmicb.2020.01435 (PMC7381354; doi:10.3389/fmicb.2020.01435)

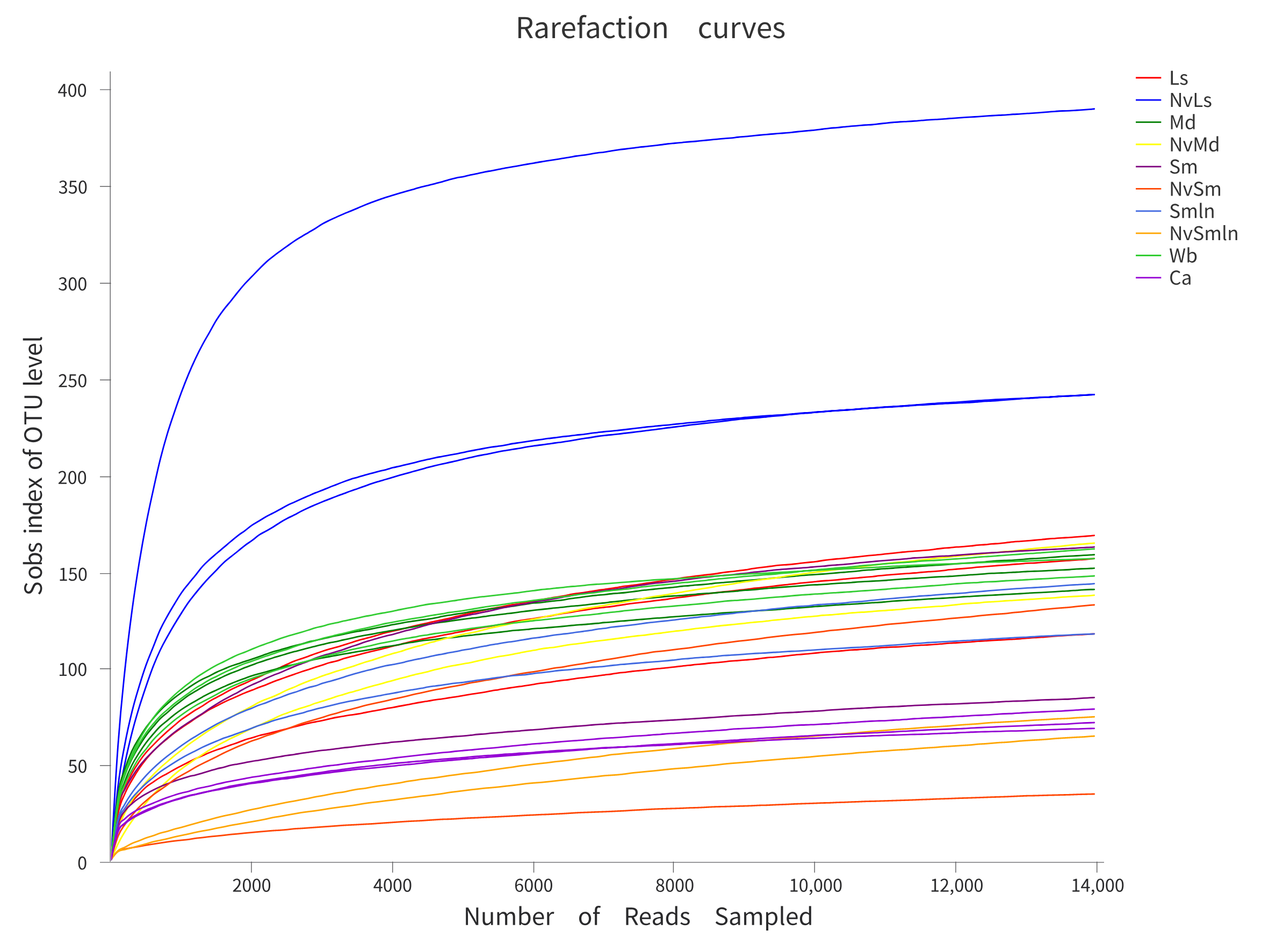

Supplement: FIGURE S1 — Sparse curve analysis of the samples. [file Image_1.JPEG]

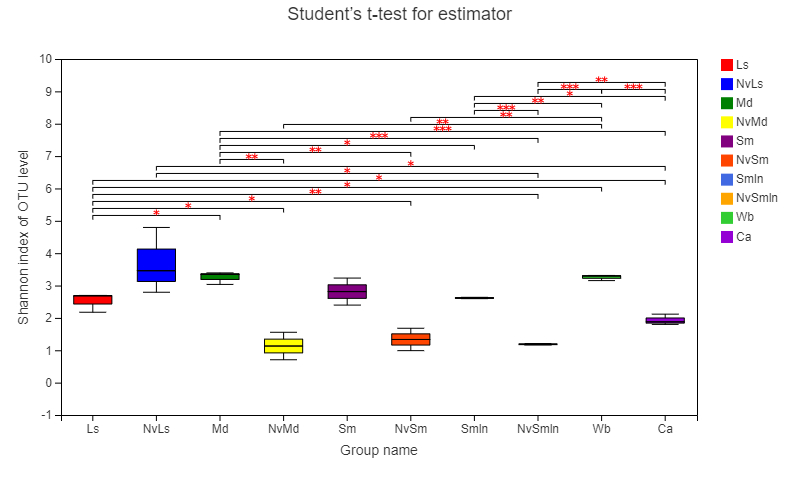

Supplement: FIGURE S2 — Shannon index at OUT level of the samples. [file Image_2.JPEG]

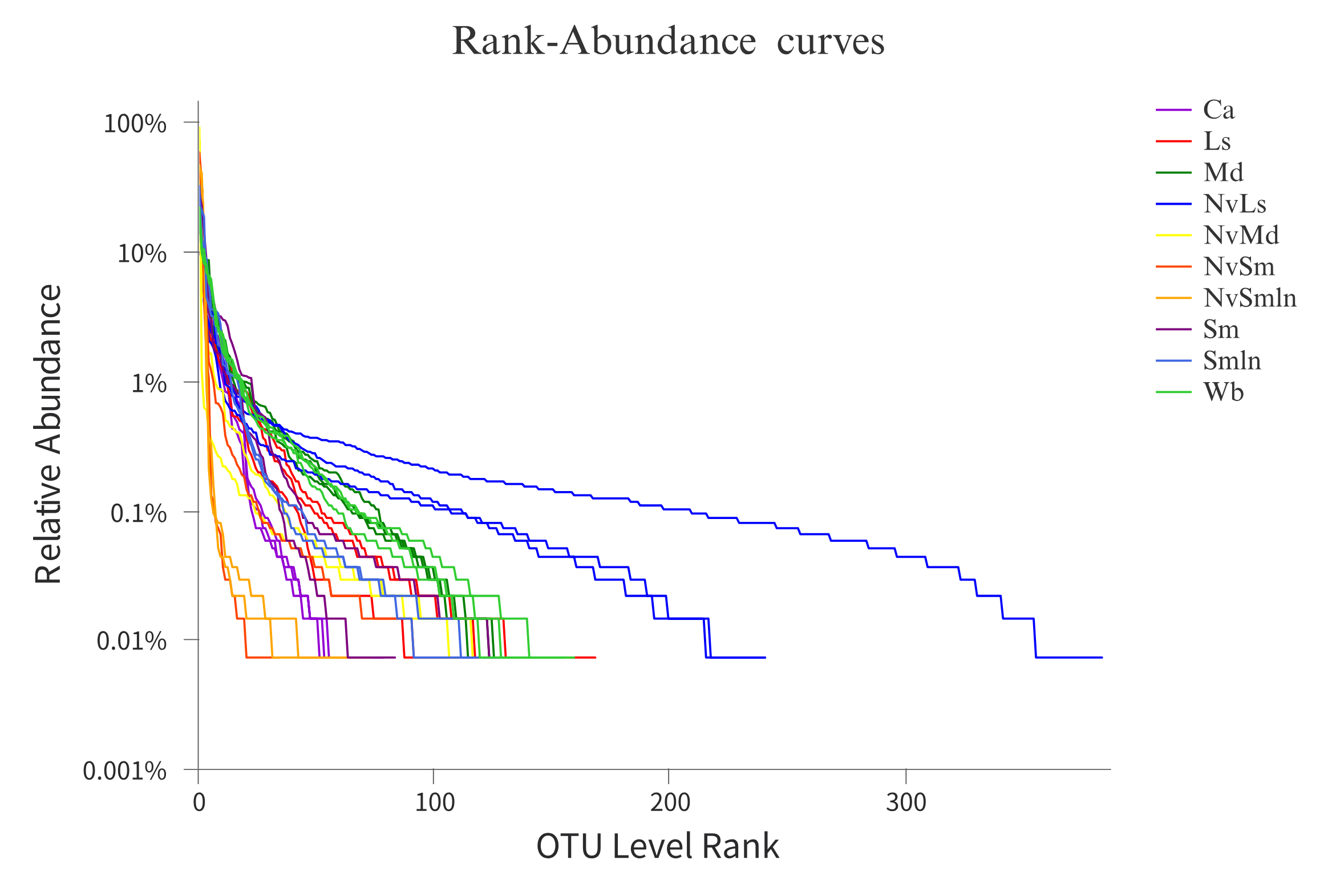

Supplement: FIGURE S3 — Rank-abundance curves at OTU level of the samples. [file Image_3.JPEG]

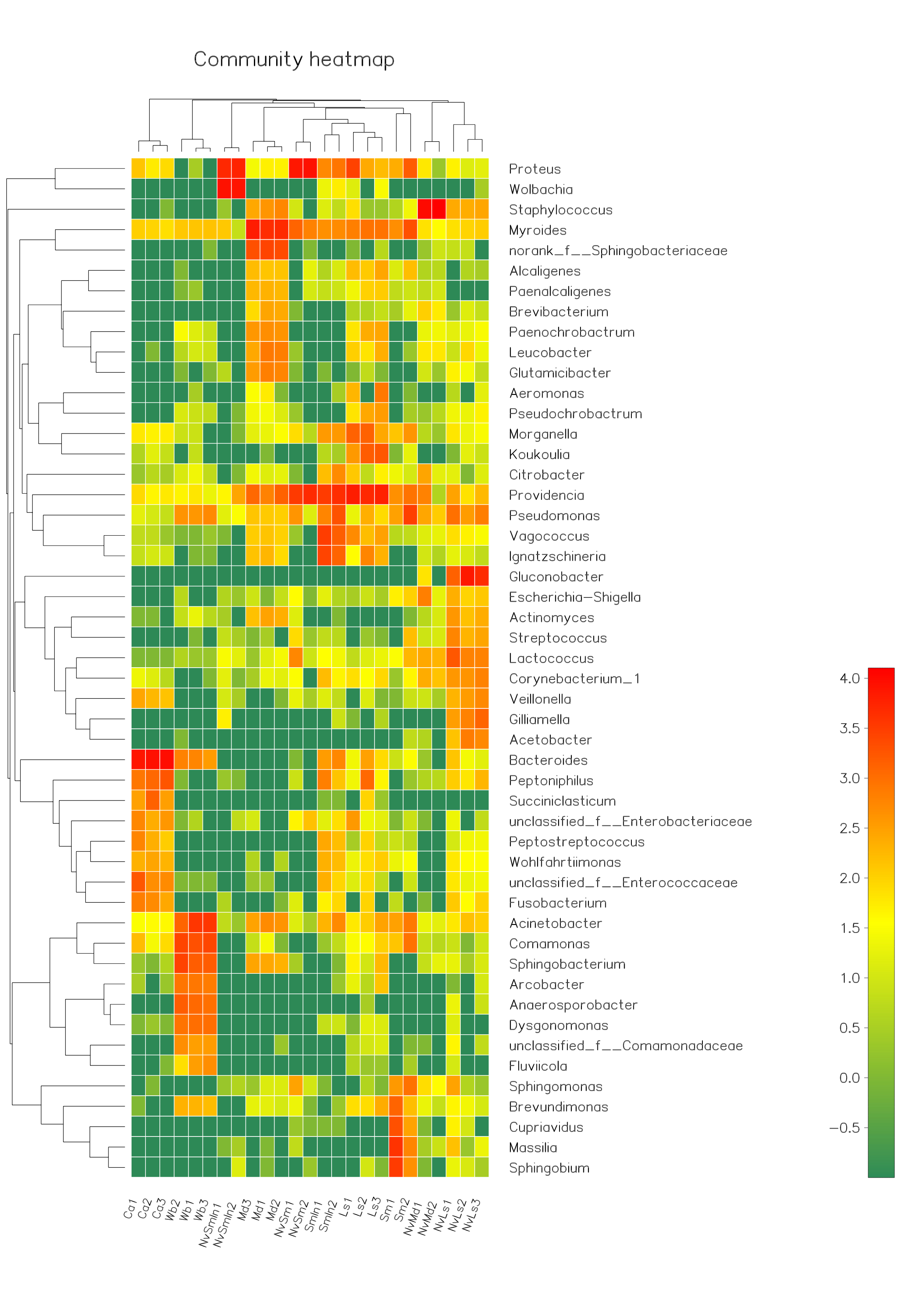

Supplement: FIGURE S4 — Relationship between Circos samples and species which can reflect the distribution ratio of dominant species in each sample through a visual circle diagram. [file Image_4.JPEG]

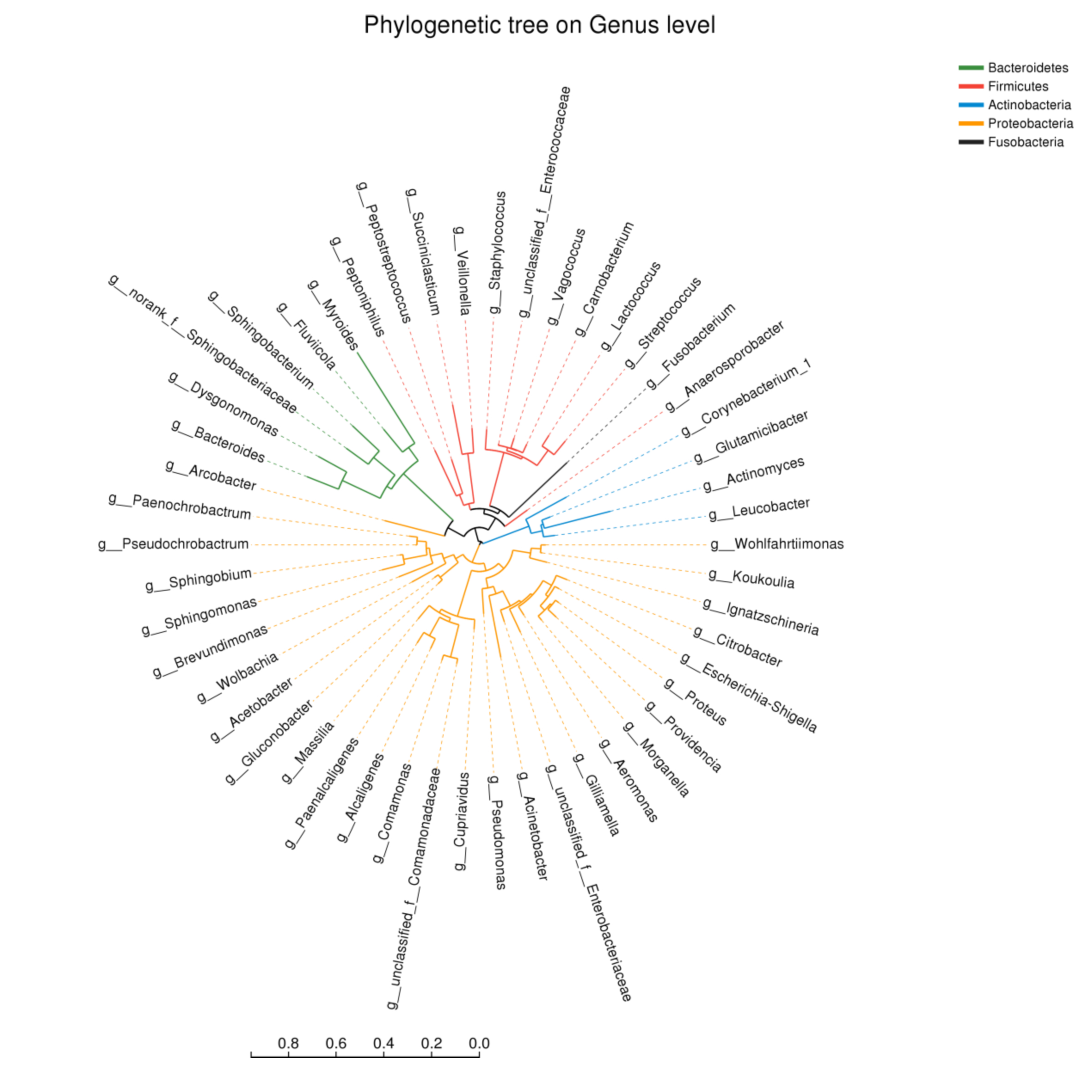

Supplement: FIGURE S5 — Heatmap analysis of the top 50 species abundance in all the samples at the genus level. [file Image_5.JPEG]

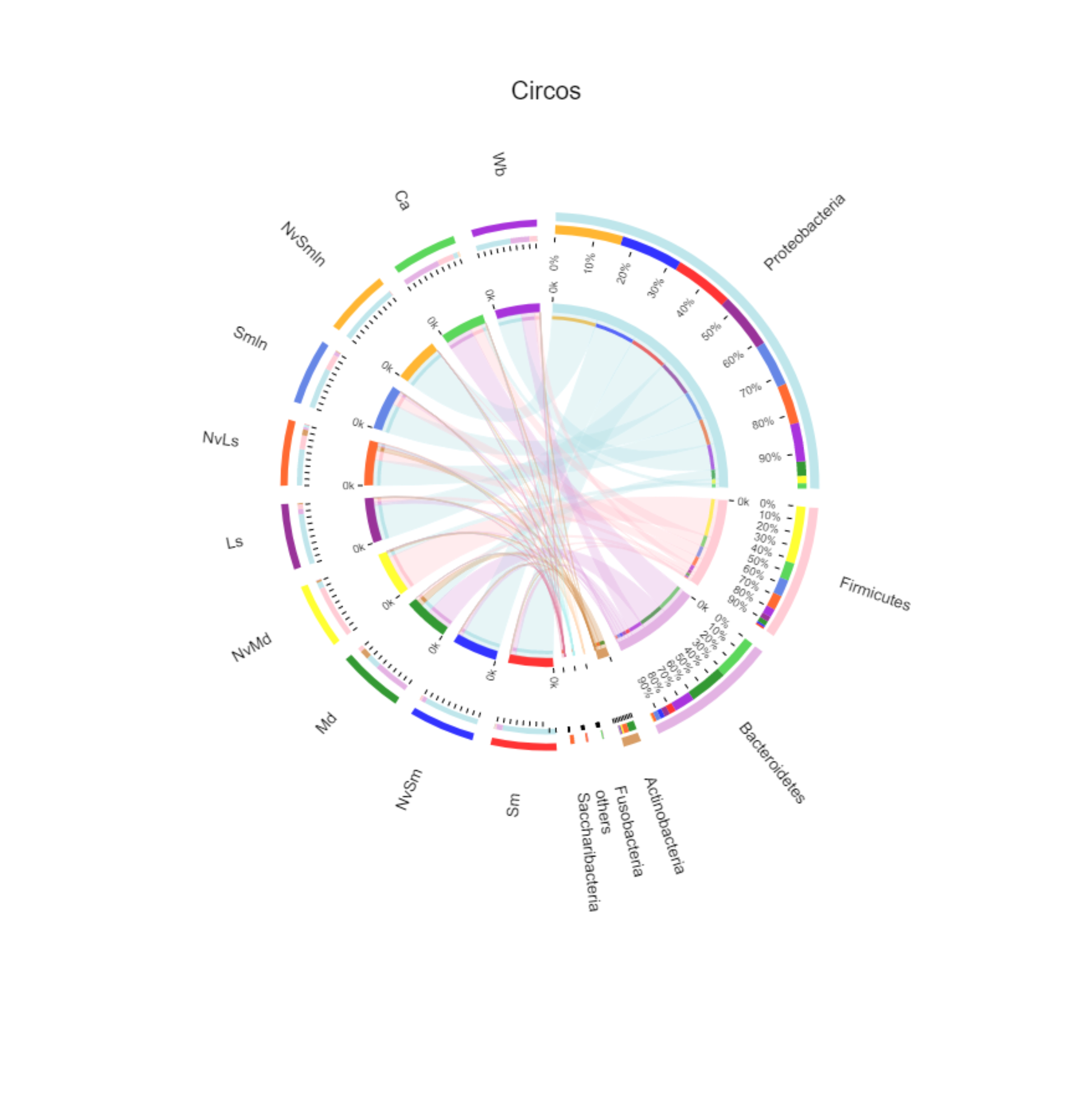

Supplement: FIGURE S6 — Cyclic phylogenetic tree of the top 50 species abundance in all samples at the genus level. [file Image_6.JPEG]

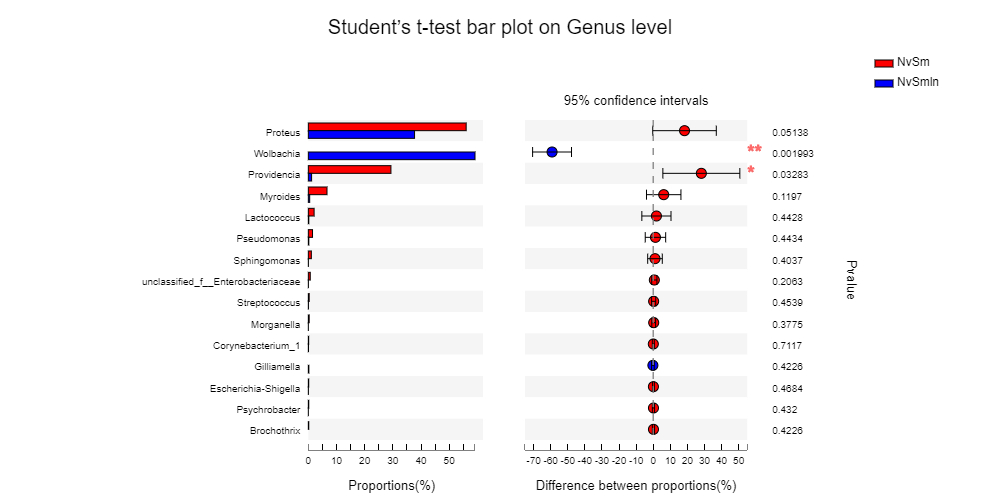

Supplement: FIGURE S7 — Student’s t-test bar plot on Genus level between two samples. [file Image_7.JPEG]
